# Decoding Early Clues: Immune Mechanisms, Prevention, Diagnosis, and Treatment of IgE-Mediated Peanut and Tree Nut Allergy in Children

**DOI:** 10.3390/biomedicines13102377

**Published:** 2025-09-28

**Authors:** Karolina Dumycz, Agnieszka Szczukocka, Maria Wawszczak, Katarzyna Grzela, Wojciech Feleszko, Marek Kulus

**Affiliations:** 1 Department of Pediatric Pneumonology and Allergy, Medical University of Warsaw, 02-091 Warsaw, Poland; 2 Doctoral School, Medical University of Warsaw, 02-091 Warsaw, Poland

**Keywords:** food allergy, peanut allergy, tree nut allergy, prevention, phenotypes, atopic dermatitis

## Abstract

The rising prevalence of food allergies, particularly to peanuts and tree nuts, poses significant challenges for pediatric health worldwide. These allergens are among the leading causes of severe allergic reactions, including anaphylaxis, often manifesting in early life. This review synthesizes the current knowledge on the immune mechanisms underlying these allergies, emphasizing the interplay between genetic, immunologic, and environmental factors in shaping allergic sensitization. Advances in prevention strategies, including early allergen introduction, have been critically evaluated. Predictive and diagnostic methodologies, from traditional IgE evaluation to cutting-edge proteomics and metabolomics approaches, have been explored to identify biomarkers that predict allergy onset and severity. By unraveling early immunological and molecular signatures, this study aimed to summarize the early prediction, prevention, diagnosis, and treatment of peanut and tree nut allergies, ultimately contributing to more effective interventions and a better quality of life for affected children.

## 1. Introduction

Peanut and tree nut allergies are among the most common and potentially severe food allergies (FA) affecting children worldwide [1]. These immunoglobulin E (IgE)-mediated allergies can cause a spectrum of clinical manifestations, ranging from mild oral symptoms to life-threatening anaphylaxis [2]. The prevalence of peanut and tree nut allergies has increased in many developed countries over the past few decades, posing significant challenges for affected individuals, families, healthcare systems, and society at large. This article provides a comprehensive overview of the current understanding of IgE-mediated peanut and tree nut allergies in children, focusing on early immunological mechanisms, risk factors, prevention strategies, epidemiology, natural history, and clinical presentations. We examined the complex interplay between genetic susceptibility, environmental exposures, and immune dysregulation that contribute to the development of these allergies. Additionally, we discuss emerging research on distinct immunological endotypes and potential biomarkers that may enable early prediction and targeted interventions. Understanding the multifaceted nature of peanut and tree nut allergies is crucial for developing effective prevention strategies, improving diagnostic accuracy, and optimizing management. This review synthesizes the latest evidence to provide clinicians and researchers with an up-to-date perspective on this important and evolving field of pediatric allergy.

## 2. Mechanisms of IgE-Mediated Food Allergy

### 2.1. General Immunopathogenesis and Pathways of Peanut and Tree Nut Allergy

FA result from the failure to establish and maintain oral tolerance to dietary antigens. The development of allergic sensitization, including to peanuts and tree nuts, is often initiated through non-oral routes, such as the skin, especially under conditions of compromised epidermal barrier function. The “outside-in” hypothesis emphasizes the central role of skin barrier dysfunction in initiating allergic sensitization. Atopic dermatitis (AD), a common chronic inflammatory skin disease of infancy, is characterized by defects in skin barrier integrity, increased transepidermal water loss (TEWL), and decreased levels of key structural proteins such as filaggrin and loricrin. These defects facilitate the penetration of environmental allergens and microbial antigens, setting the stage for epicutaneous sensitization. Studies in both humans and mouse models have shown that epicutaneous exposure to food allergens, particularly in the context of skin inflammation, leads to a robust Th2 response and the generation of food-specific IgE [3,4]. In contrast, the “inside-out” hypothesis assumes that a dysregulated Th2 response is responsible for dysfunction of the barrier function that leads to subsequent allergen penetration and development of allergic sensitization.

**Figure 1:**
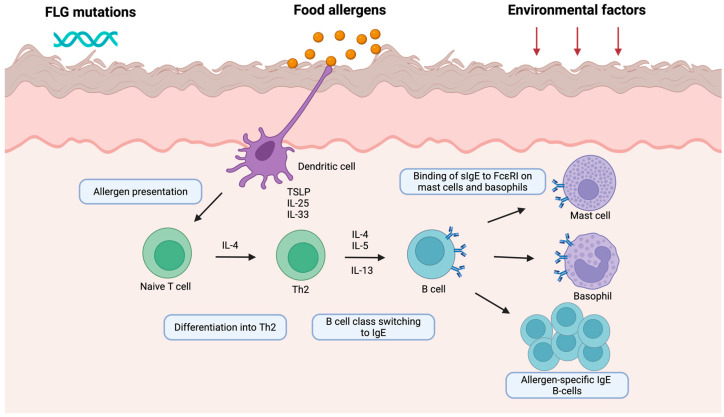
Epicutaneous sensitization pathway. Food allergens penetrate the skin barrier and are taken up by dendritic cells, which, under the influence of IL-25, IL-33, and TSLP, promote Th2 polarization and the secretion of IL-4, IL-5, and IL-13. These cytokines drive B cell class switching to allergen-specific IgE, which binds to FcεRI on mast cells and basophils, priming them for activation upon re-exposure to the allergen. Created in BioRender. Dumycz, K. (2025) https://BioRender.com/tan3wmm.

The process begins with the uptake of environmental food allergens by skin-resident dendritic cells, which migrate to draining lymph nodes and present antigens to naïve CD4^+^ T cells. In genetically susceptible individuals, this antigen presentation skews the immune response towards a Th2 phenotype, characterized by the production of cytokines such as interleukin (IL) 4, IL-5, and IL-13. These cytokines promote class switching of B cells to produce allergen-specific IgE antibodies, which subsequently bind to high-affinity FcεRI receptors on mast cells and basophils (Figure 1). Upon re-exposure to the allergen, cross-linking of bound IgE leads to degranulation of these effector cells and the release of histamine and other inflammatory mediators that drive the clinical symptoms of allergic reactions [5]. In murine models of FA, sensitization through different barriers has been extensively studied [6,7]. In mice epicutaneously sensitized by repeated application of ovalbumin (OVA), expansion of mast cells in the jejunum was observed, and oral food challenge (OFC) resulted in the induction of an anaphylactic reaction accompanied by an increase in IL-4 serum concentration [8]. Epicutaneous sensitization followed by FA development to OVA and peanuts is facilitated by IL-25, IL-33, and TSLP, which are critical drivers of Th2-related responses [9,10,11,12]. Specifically, mouse models of peanut allergy revealed that repeated epicutaneous exposure to peanut oil resulted in increased specific IgE (sIgE) and leukocyte and eosinophil counts, as well as increased IL-13 levels [13]. Interestingly, in a mouse model developed to mimic the LEAP (Learning Early About Peanut allergy) study, sensitization and FA development were an implication of exposure to peanut allergens through the skin and airways. On the other hand, ingestion of peanut butter protected mice against FA development [14]. In addition to skin, solely airway exposure to peanut allergens accompanied by airborne pollutants (such as particulate matter and diesel exhaust particles) can induce peanut-dependent anaphylaxis upon oral ingestion [15,16,17]. Consistently, experimental studies have demonstrated that cutaneous exposure to tree nut allergens leads to sensitization and subsequent development of clinical allergy, as confirmed by positive OFC [18,19,20,21,22]. A study by Lack et al. demonstrated a link between peanut allergy and the use of topical skin products containing peanut-derived ingredients [23]. Fox et al. observed a higher incidence of FA in households in which peanuts were frequently consumed [24]. Brough et al. provided further evidence of a dose-dependent relationship between household dust peanut protein levels and the risk of peanut sensitization and allergy in infants [25]. This association was particularly pronounced in children with compromised skin barrier function, either due to filaggrin (*FLG*) loss-of-function mutations resulting in reduced FLG expression, in those with AD, or in those with pre-existing egg allergy [25,26].

### 2.2. Distinct Endotypes in Children with Peanut and Tree Nut Allergy

Children with FA present different immune, genetic, or metabolic endotypes compared to individuals without FA. Recent studies have begun to uncover distinct immunological endotypes in children with peanut and tree nut allergy [27]. A study by Neeland et al. compared the immune profiles of one-year-old infants with peanut allergy, peanut-sensitized but tolerant, and non-allergic controls. Peanut allergic infants exhibited higher frequencies of CD19^high^HLA-DR^high^ B cells and increased production of TNF-α upon stimulation, indicating heightened immune activation. Peanut sensitized but tolerant infants had greater levels of plasmacytoid dendritic cells and IL-2-producing naive CD4^+^ T cells; however, both peanut allergic and peanut sensitized but tolerant groups showed reduced IFN-γ expression in effector memory T cells, suggesting an impaired Th1 regulatory axis [28]. Zhou et al. revealed that PBMCs from peanut-allergic children produce elevated levels of pro-inflammatory cytokines, including MCP-1, MIP-1α, IL-6, and IL-10, in response to peanut protein stimulation. These children also showed increased differentiation of CD11c^+^CD209^+^ dendritic cells, which were dependent on IL-4/IL-13 signaling and interacted reciprocally with allergen-specific Th2 cells, reinforcing the allergic endotype [29]. Transcriptomic profiling studies have further elucidated immune dysregulation in children with nut allergies. RNA sequencing performed by Lee et al. demonstrated the upregulation of gene modules related to type I interferon signaling and cytokine production and the downregulation of modules involved in humoral immunity. These transcriptomic changes were associated with increased neutrophil and decreased regulatory T cell frequencies, highlighting the complexity and heterogeneity of immune responses in nut allergy [30]. Skin-focused studies have also provided important insights. Leung et al. showed that non-lesional skin from children with AD and peanut allergy had higher TEWL, reduced levels of FLG and sphingolipids, increased colonization with Staphylococcus aureus, and upregulated expression of hyperproliferative keratins (K5, K14, and K16). Transcriptomic analysis has revealed the enrichment of dendritic cell markers and type 2 immune pathways [31]. What is more, the proteomic analysis of children with AD and peanut allergy revealed that the non-lesional skin of these individuals is characterized by increased levels of proteins associated with inflammatory response (S100 proteins, alarmins, and protease inhibitors), glycolysis, and antioxidant defense enzymes [32]. Different immune subtypes and children allergic to nuts are summarized in Table 1. These findings indicate that the skin of individuals with AD and peanut allergy exhibits a distinct profile characterized by impaired barrier function and heightened immune activation.

## 3. Risk Factors and Early Prediction of Peanut and Tree Nut IgE-Mediated Food Allergy Development

Numerous studies have identified risk factors associated with the development of peanut and tree nut allergies in early life. Since FA development is generally facilitated by exposure of the skin, especially with an impaired barrier, to environmental allergens, the presence of AD, particularly early-onset and severe forms, is a strong predictor of subsequent FA [33,34]. The T-CHILD study demonstrated that children with both AD and allergic sensitization had significantly higher odds of developing FA compared to non-sensitized children without AD [35]. In the PASTURE study, early-transient (OR 3.8 (2.02–7.13)) and early persistent AD phenotypes (OR 7.8 (4.413–14.72)) were strongly associated with FA at the age of 6 years [36]. Similarly, in the Generation R study and in the Japan Environment and Children (JETS) Cohort, early-transient and persistent AD were associated with FA development [37,38].

Genetic susceptibility plays a central role in modulating risk. Loss-of-function mutations in the *FLG* gene, which encodes the key epidermal barrier protein filaggrin, are strongly associated with AD and peanut allergy [39]. Other genes within the epidermal differentiation complex, such as *FLG2*, *HRNR*, and *TCHH1*, have also been implicated. Data from the LEAP (Learning Early About Peanut Allergy) study showed that *FLG* variants were correlated with greater AD severity and increased odds of peanut allergy, independent of baseline eczema severity [40]. Furthermore, numerous studies have revealed that genetic variants of *HLA*, *IL13*, *SPINK5*, *SERPINB*, and *C11orf30* are associated with FA development [41]. Children who developed a peanut allergy had the HLA-DQA1 *01:02 genetic variant [42]. What is more, *MALT1* and *rs57265082* (*MALT1 SNP*) were associated with the development of peanut allergy in individuals who avoided peanuts in the LEAP study [43]. Genomic and epigenomic variations exhibit different patterns in patients with FA. Peanut allergic children were characterized by methylation of a 16 CpG site panel [44]; however, methylation of a panel of 203 CpG sites was associated with severity of reaction to peanuts [45]. Interestingly, a study conducted in twins showed that genetic factors play a significant role in the development of peanut, pistachio, and walnut allergy, since the concordance between twins was significant [46].

Apart from risk factors, the identification of markers that precede FA development is essential for the close monitoring of such children. In a study of a general population-derived birth cohort, the authors found that in infants who developed FA, cord blood displayed a higher monocyte to CD4^+^ T cell ratio and a lower proportion of natural regulatory T cells (nTreg) in relation to the duration of labor. CD14^+^ monocytes of food-allergic infants secreted higher amounts of inflammatory cytokines (IL-1β, IL-6, and tumor necrosis factor-α) in response to lipopolysaccharide [47].

Another study evaluated the stratum corneum of infants before the development of any allergic disease. In the stratum corneum of children with future FA or AD with concomitant FA, the absolute amounts of unsaturated (N24:1) (C18-sphingosine) and (N26:1) (C18-sphingosine) ceramide were increased compared to those in healthy children. Children with future AD had normal levels of these molecules. IL-33 levels were upregulated in infants with future FA. Logistic regression analysis revealed strong FA predictive power for the combination of dysregulated lipids and cytokines, with an odds ratio of 101.4 (95% CI = 5.4–1910.6) [48].

The identification of risk factors in infancy is crucial for monitoring children at an increased risk of developing FA and implementing early preventive strategies, particularly in this vulnerable population.

## 4. Peanut and Tree Nut IgE-Mediated Food Allergy Prevention Strategies

Effective prevention of peanut and tree nut allergy in early childhood hinges on two primary approaches: enhancement of skin barrier function and induction of oral tolerance through early dietary introduction. The “dual allergen exposure hypothesis” suggests that while cutaneous exposure leads to sensitization, oral exposure fosters tolerance [49].

In Israel, where peanut-based snacks are commonly introduced early in infancy, the prevalence of peanut allergy is markedly lower than in countries such as the UK, where avoidance had been the standard practice [50].

The LEAP trial was a landmark study demonstrating that the early introduction of peanuts in high-risk infants aged 4–11 months reduced the incidence of peanut allergy by over 80% compared to avoidance [23]. The protective effect was sustained in the LEAP-On study, even after a year of peanut avoidance from 5 to 6 years of age. These findings have prompted revisions to international guidelines. The early introduction of peanuts, especially in high-risk populations such as infants with moderate-to-severe eczema or egg allergy, has been considered as a recommendation [51,52].

Subsequent EAT study has corroborated these findings and expanded the evidence to different population. In exclusively breastfed infants without risk factors for allergy development, early peanut introduction resulted in lower rates of peanut allergy development in a per-protocol analysis [53].

However, a recent population-based study conducted by Soriano et al. in Australia demonstrated that, although peanut introduction in the first year of life increased more than threefold (from 21.6% to 85.6%) from 2007 to 2018 (before and after early introduction guidelines), there was only a non-significant decrease in peanut allergy in the population over this period [54]. Abrams et al. suggested that additional factors may contribute to the development of peanut allergy. The authors concluded that the regularity of ingestion may play as significant a role as the timing of introduction [55].

There is scarce data on whether the early introduction of tree nuts can prevent the development of tree nut allergy. In one study, based on the HealthNuts cohort, the association between cashew introduction before 1 year of age and a reduced risk of cashew allergy was evaluated [56]. There was weak evidence that the early introduction of cashews was associated with reduced odds of cashew allergy (adjusted OR, 0.19; 95% CI, 0.00–1.09; *p* = 0.07) [57]. This was the first report showing that the early introduction of tree nuts may also be beneficial in FA prevention.

Barrier-enhancing strategies, particularly the use of emollients to reduce AD and FA, have yielded mixed results in clinical trials [58]. Standard petroleum-based emollients have failed to reduce AD and FA development, as shown in two large clinical trials: BEEP and PreventADALL [59,60]. Moreover, emollients used in the general population may increase the risk of FA development, probably through transcutaneous sensitization [61].

In contrast, a study on ceramide-rich trilipid emollient usage showed that regular use may lower TEWL and allergen-specific IgE levels more effectively than petroleum-based emollient [62]. In another study, high-quality emollient reduced the risk of AD development in high-risk infants (32.8% in the intervention group vs. 46.4% in the control group) [63].

Once AD is developed, early anti-inflammatory interventions may be beneficial. The PACI study has provided evidence that anti-inflammatory management of AD may reduce the subsequent risk of FA (egg allergy); however, further research is needed to evaluate FA to different allergens [64].

## 5. Prevalence of Peanut and Tree Nut IgE-Mediated Food Allergy in Children

### 5.1. Peanut Allergy

Peanut remains one of the most prevalent food allergens globally, with its prevalence varying according to dietary practices and environmental exposures such as pollen. The highest rates are observed in the U.S, Canada, and Australia, where prevalence estimates range from approximately 0.6% to 2% [65]. In the U.S. alone, parent-reported data suggest that approximately 1.6 million children are affected by peanut allergy [66,67]. In Europe, the overall lifetime and point prevalence of self-reported peanut allergy are estimated to be 1.5% and 2.1%, respectively, whereas the point prevalence of food challenge–confirmed allergy is approximately 0.1% [68]. Although the prevalence of peanut allergy varies across Europe, it is generally higher in Western and Northern European countries than in Southern and Eastern Europe, with the UK cited as having among the highest rates. In many Asian countries, peanut allergy appears to be less frequent and is often not included among the leading food allergens [69].

The burden of peanut allergy in children has increased in certain countries, particularly in the U.S. and the UK, over the past decade. A national U.S. claims database analysis revealed that the annual incidence in one-year-old children rose from 1.7% in 2001 to 5.2% in 2017 [70]. A nationwide study in the UK based on clinician-documented diagnoses reported that the prevalence of peanut allergy doubled between 2001 (0.24 per 1000 individuals) and 2005 (0.51 per 1000 individuals) [71]. Furthermore, a retrospective review of UK medical records spanning three decades demonstrated that the point prevalence per 100,000 individuals increased from 31 to 202 in the general population and from 116 to 635 in children between 2000 and 2015 [72]. This study also documented that the overall incidence of peanut allergy in the UK more than doubled during this period, from 8.6 to 18.2 per 100,000 individuals. Canadian data suggest a more stable trend, with the study reporting no significant change in peanut allergy prevalence in Montreal [73] between 2000 and 2007 (from 1.34% to 1.62%), but a tendency towards an increase in the Canadian nationwide study between 2010 and 2017 (from 2.7% to 3.5%) was observed [74]

In addition, similar to the U.S. and the UK, data from Australian studies indicate a trend towards an increased prevalence of peanut allergy. A retrospective cohort study of children aged 0–6 years in the Australian Capital Territory reported an increase in incidences of peanut allergy from 0.73% in children born in 2001 to 1.15% in children born in 2004 [75].

### 5.2. Tree Nut Allergy

Tree nut allergy is on the rise, largely due to the widespread use of these nuts as snacks and their presence as hidden ingredients in processed food. In the U.S., the estimated prevalence of tree nut allergy is approximately 0.4% to 1.1%, affecting around 0.9 million children, while in Europe, prevalence rates range more broadly from 0.03% to 8.5% [66,76].

An analysis (Food Allergy and Anaphylaxis Network (FAAN)) of over 5000 individuals in the U.S. found that the most common causes of FA were walnuts (34%), cashews (20%), almonds (15%), pecans (9%), and pistachios (7%), with hazelnuts, Brazil nuts, and macadamia nuts reported less frequently (each below 5%) [77]. In Europe, hazelnut allergy is the most prevalent, primarily due to cross-reactivity with birch pollen, whereas pistachio allergy appears to be the least common [78]. In Australia, cashew allergy predominates, with clinically confirmed tree nut allergy affecting up to 3.0% of children at age 6 and 2.3% of those aged 10–14 years [79,80,81]. Tree nut allergy, like peanut allergy, appears to be less common in many Asian countries [79,82].

Evidence on trends in tree nut allergy prevalence over time is limited. A national telephone survey conducted at three time points in the U.S. (1997, 2002, and 2008) found no significant change in adult self-reported tree nut allergy but reported an increase among children, from 0.2% in 1997 and 2002 to 1.1% in 2008 [77]. Contrary Gupta et al. reported that the overall prevalence remained relatively stable, with rates of 1.0% in 2009–2010 and 1.2% in 2015–2016 [83].

### 5.3. Peanut and Tree Nut Co-Allergy

The coexistence of peanut and tree nut allergy has frequently been reported in self-reported studies, with estimated rates ranging from 20% to 60% [80]; however, clinical data suggest that the true prevalence of confirmed co-allergy is lower. A retrospective study conducted in the UK found that among 94 children with confirmed peanut allergy, only 7.4% were also clinically allergic to one or more tree nuts [84]. Similarly, a study from the U.S. reported that while 65% of peanut-allergic patients showed sensitization to tree nuts, only 20% were clinically allergic [85]. In another cohort, 86% of peanut-allergic individuals were sensitized to tree nuts, but only 34% were confirmed to have an allergy to at least one tree nut [86].

Australian studies have shown comparable results. Population-based data from the HealthNuts study found that 40% of children with peanut allergy at age six had a confirmed tree nut allergy [81]. These results were supported by the SchoolNuts study, which identified that approximately 30% of adolescents aged 10–14 years with peanut allergy were also allergic to one or more tree nuts, based on clinical history, sensitization testing, and OFC [79].

In a study conducted in Central Europe (Poland), 35% of children sensitized to peanuts were also sensitized to walnuts [87]. Moreover, among children with suspected FA, 50% and 18% of individuals were sensitized to at least two and three different nuts, respectively [87,88].

Despite the structural similarities between some allergenic proteins, peanuts and tree nuts belong to distinct botanical families [89]. Studies using IgE inhibition assays and serological analyses indicate that peanut and tree nut allergies do not frequently result from cross-reactivity but are more likely to develop independently [90]. Consequently, peanut allergy should not automatically lead to the avoidance of all tree nuts without proper evaluation. Clinical evidence suggests that the actual co-allergy rate is under 35%, and the relationship between peanut and tree nut allergy is highly individual and not reliably predicted by sensitization alone [91].

### 5.4. Tree Nut Co-Allergy

There is limited but growing data on the coexistence of allergy to different tree nuts, showing that their prevalence ranges from 12% to 47%. The comparable results were obtained from U.S. studies reporting the prevalence of tree nut co-allergy ranging from 12% to 37% [77,91,92]. Even higher rate of incidents has been shown in the HealthNuts cohort, where 47% of children with a tree nut allergy had allergy to more than one nut [81].

Tree nut sensitization and allergy often increase with age. For example, one study showed that by age two, 19% of children were sensitized to multiple tree nuts, and 2% were allergic to more than one. By age 14, these numbers increased significantly, with 86% showing multisensitization and 47% having clinical allergy to multiple tree nuts [93].

Cross-reactivity among tree nuts is often associated with shared botanical families and common allergenic components, such as storage proteins and panallergens. The most robust cross-reactivity was observed between walnuts and pecan nuts and between cashew and pistachios. Walnut also shows some cross-reactivity with Brazil nut and hazelnut, while moderate in vitro cross-reactivity has been noted among hazelnut, cashew, Brazil nut, pistachio, and almond [91,94,95].

The NutCracker study from Israel, which prospectively assessed 83 children with tree nut allergy, reported that although most patients were sensitized to 5–6 tree nuts, more than half were allergic to only 1–2 nuts. The highest rates of clinical allergy among sensitized individuals were observed for walnuts (74.6%) and cashews (65.6%). Approximately two-thirds of walnut-allergic patients were also allergic to pecan nuts, and a similar proportion of cashew-allergic children reacted to pistachios. Notably, all pecan- and pistachio-allergic patients were also allergic to walnuts and cashews, respectively [96]. Another study of children with multiple FA confirmed that pecan-walnut and cashew-pistachio co-allergies are frequent, with all pistachio-allergic children failing cashew OFC [90]. A multicenter European study evaluating peanut, tree nut, and sesame co-allergy also found that 60.7% of participants were allergic to more than one nut or seed, with clear clustering observed between cashew-pistachio and walnut-pecan, as well as co-occurrence with hazelnut and macadamia nut allergies [97]. However, the findings of the HealthNuts study suggest that such clustering may not be universal. For example, only 36% of children with cashew allergy had coexisting pistachio allergy, and walnut-pecan co-allergy was not as common as in other cohorts [81]. These differences may reflect variations in dietary habits, genetic predisposition, or allergen exposure across populations. Prevalences to different nuts are summarized in Table 2.

## 6. Natural History

### 6.1. Peanut IgE-Mediated Allergy

Findings from a study reported by Skolnick et al. suggested that approximately 20% of children eventually outgrow peanut allergy [98]. More recent data indicate that peanut allergy resolves in around 33.9% of children by the age of 10 years, with the majority achieving tolerance by age 6 years [99]. Key immunological markers associated with resolution include a decline in Ara h 2-specific IgE levels and increases in peanut-specific IgG4, Ara h 2-specific IgG4, and the ratios of IgG4 to IgE for both peanut and Ara h 2. Of these, the most predictive marker was peanut-specific IgE measured at 1 year of age [99]. The HealthNuts study evaluated the natural course of peanut and egg allergy development. Compared to egg allergy, peanut allergy showed a lower rate of resolution before the age of 6 years. Factors at age 1 associated with persistent peanut allergy included a skin prick test (SPT) wheal size of ≥8 mm, sensitization to tree nuts, and early-onset severe eczema [56]. An integrative analysis of data from three major cohorts, EAT (Enquiring About Tolerance), LEAP, and PAS (Peanut Allergy Sensitization), revealed that 56.3% of children with peanut allergy developed the condition by 12 months of age, while approximately 32% experienced an early resolution. The rates of early peanut allergy resolution varied across cohorts, with 54.2% in the EAT study, 41.4% in the LEAP study, and 18.6% in the PAS study [100].

### 6.2. Tree Nut IgE-Mediated Allergy

Our current understanding of the natural history of tree nut allergy is primarily based on retrospective clinical reviews and self-reported survey data, which significantly limit the depth and accuracy of the available evidence. Data suggest that a subset of children with tree nut allergy may eventually develop tolerance; however, reported resolution rates range between 9% and 14%, which appears to be lower than those observed for peanut allergy [80]. Fleischer et al., in a retrospective analysis of 278 children diagnosed with tree nut allergy based on clinical history or sensitization (positive SPT or sIgE), showed the rate of resolution confirmed by negative oral food challenge at 9%. Persistent allergy was more common in children with AD and other coexisting FA, whereas the initial reaction type and comorbid asthma or allergic rhinitis did not significantly influence persistence [92]. Even higher rate of tree nut allergy resolution (14% of participants) was observed in a cross-sectional population-based survey in a study by Gupta et al. [67]. Similarly, a clinical presentation including a history of severe reactions was not associated with the persistence of tree nut allergy.

A population-based cohort study in Sweden assessed the prevalence of tree nut sensitization and allergy symptoms in young adults. Sensitization accompanied by allergic symptoms was present in 7.9% of the participants, or 4.3% when excluding mild oral allergy symptoms. Atopic conditions in early childhood, such as egg allergy, AD, and asthma, were associated with the development of tree nut symptoms and storage protein sensitization at 24 years of age. Current eczema and markers of asthma severity were identified as risk factors for persistent tree nut allergy in young adults [101].

### 6.3. Peanut and Tree Nut IgE-Mediated Allergy

Data from a large cross-sectional population-based survey in the U.S. have provided insights into the natural history of self-reported FA, including peanut and tree nut allergies. Children with concurrent peanut (15.6%) and tree nut (14.3%) allergies exhibited significantly lower tolerance development rates. Several factors were associated with a higher likelihood of outgrowing FA, including mild to moderate reactions, being allergic to only one food, presenting with eczema as the only symptom, and White compared to Black ethnicity [102]. Furthermore, the probability of developing tolerance was higher when the first allergic reaction occurred at a younger age and decreased when the initial reactions occurred later in life, regardless of the specific allergen, severity, or clinical presentation [103].

## 7. Clinical Manifestations of IgE-Mediated Reactions to Peanuts and Tree Nuts

Most allergic reactions to peanuts and tree nuts are mediated by IgE antibodies. These reactions range from mild symptoms, such as urticaria or pollen-associated oral allergy, to severe manifestations, including life-threatening anaphylaxis [104].

### 7.1. Localized Reactions

Contact with food allergens, such as peanut butter, typically results in localized symptoms confined to the site of skin exposure and rarely leads to systemic reactions [105,106,107,108,109]. Accidental eye exposure, often due to touching the eye with food-contaminated hands, can cause notable periorbital swelling, redness, and itching. Occasionally, exposure via saliva through kissing, sharing utensils, or straws may induce either localized or systemic allergic symptoms, although this is uncommon [110,111].

### 7.2. Pollen-Food Allergy Syndrome (PFAS)

PFAS occurs due to cross-reactivity between pollen allergens and structurally similar proteins in raw plant-derived food. It typically results in mild, self-limited symptoms restricted to the oral cavity and pharynx [112]. The risk of anaphylaxis associated with PFAS is generally low [113]. Individuals sensitized to birch pollen (particularly Bet v 1, a PR-10 protein) may develop PFAS in response to peanuts or tree nuts due to exposure to homologous proteins such as Ara h 8 (peanut), Cor a 1 (hazelnut), or Jug r 5 (walnut). The reported prevalence of PFAS among pollen-allergic individuals ranges from 4.7% to 20% [114,115]. Lipid transfer proteins (LTPs) are another class of cross-reactive plant allergens implicated in peanut and tree nut allergy. The primary sensitizer in LTP syndrome is Pru *p* 3 from peach. Unlike PR-10 proteins, LTPs are heat-stable and resistant to enzymatic degradation, making them more likely to cause systemic and potentially severe allergic reactions. LTP syndrome is more prevalent in Mediterranean countries, whereas regions with high birch pollen exposure tend to show lower rates of LTP-related allergy [115,116].

### 7.3. Anaphylaxis

Peanut and tree nut allergies are among the leading causes of severe and fatal allergic reactions in both children and adults [117]. Sicherer et al. evaluated 122 children who had experienced acute allergic reactions to peanuts only (68 children), tree nuts only (20 children), or both peanuts and at least one tree nut (34 children) [118]. The findings revealed that initial reactions typically occurred at home, with a median age of onset of 24 months for peanut allergy and 62 months for tree nut allergy. Symptoms developed rapidly following exposure, and skin involvement was most common, occurring in 89% of cases, of which 39% were limited to cutaneous symptoms. Respiratory symptoms were reported in 52% of reactions, and gastrointestinal involvement in 32%. Epinephrine was required in 20% of cases. Despite education on allergen avoidance, accidental exposures were frequent: 56% of children with peanut allergy and 30% with tree nut allergy experienced unintentional ingestion during a median follow-up of 5.5 years, averaging two incidents per child. The clinical manifestations during accidental exposures were generally similar to those observed during the initial reaction. Unlike initial reactions, which occurred primarily at home, accidental exposures were more likely to occur at school, home, or restaurants. Common routes of unintended ingestion included shared food, hidden ingredients, and cross-contamination. These findings underscore the early onset, frequent recurrence, and often severe nature of allergic reactions to peanuts and tree nuts in children. In the U.S., peanut and tree nut allergies were associated with the highest annual rates of emergency department visits for food-induced allergic reactions among children in 2014, with 5.85 and 4.62 visits per 100,000 individuals, respectively [119]. Similarly, data from the European Anaphylaxis Registry identified peanut as the most frequent food trigger of anaphylaxis in both children (26.3%) and adolescents (18.3%) [120]. This pattern was mirrored in the Australian SchoolNuts Study, which assessed 547 adolescents aged 10–14 years with suspected FA. Among these, peanut was the leading cause of anaphylaxis, accounting for 38.6% of confirmed and 30.6% of unconfirmed episodes, which is higher than any other food allergen [121]. Peanut allergy is a predominant cause of life-threatening anaphylaxis in the pediatric population, while hazelnuts and cashews rank fourth and sixth, respectively, among the most common triggers of severe anaphylactic episodes [122]. In the U.S., survey data show that 59.2% of individuals with peanut allergy and 56.1% with tree nut allergy have experienced at least one severe allergic reaction [102].

### 7.4. Allergic Reactions from Inhalation Exposure

Although rare, inhalation of peanut or tree nut proteins can provoke allergic reactions, including respiratory symptoms and occupational asthma. Exposure may occur through airborne particles, such as peanut dust or flour, or aerosolized proteins during cooking. Importantly, the aroma of peanut butter alone does not pose a risk, as it contains volatile organic compounds but no allergenic proteins [105]. Sicherer et al. reported 42 allergic reactions to peanuts or tree nuts that developed on commercial airliners after inhalation exposure. These reactions were generally mild and developed in 14 of these subjects when more than 25 individuals around them were eating peanuts served by the airlines. Medications were administered in-flight to 19 subjects, including epinephrine to five subjects [118].

## 8. Diagnosis

An accurate diagnosis of nut allergy begins with a comprehensive clinical evaluation, emphasizing a detailed history of immediate-type allergic reactions following food ingestion, as well as the identification of individual risk factors and relevant cofactors [104,123]. The second step in the diagnostic process involves confirming IgE sensitization using two primary methods: skin prick tests (SPT) with commercial extracts or fresh food, and serum-specific IgE (sIgE) measurement.

The importance of component-resolved diagnostics (CRD) is increasingly recognized, as testing for specific IgE to major allergenic proteins, such as Ara h 2 in peanuts, can offer more precise insights into the likelihood of clinical reactivity and the potential severity of allergic reactions [104]. The clinical history should guide the selection of allergens for testing; the use of broad allergen panels is not recommended. In selected cases, multiplex testing of allergenic components may be useful, particularly in identifying cross-reactions in patients allergic to multiple foods or pollen, or in those with recurrent anaphylaxis [104].

In addition to component-specific IgE testing, it is recommended to assess IgE to whole extracts, as sensitization to minor allergenic components potentially missed by CRD may still be clinically relevant. CRD offers greater specificity compared to extract-based IgE testing, enabling more accurate confirmation of allergy in patients with unclear histories or ambiguous SPT or extract-based IgE results. In pollen-allergic individuals, CRD also allows differentiation between clinically significant sensitization and cross-reactivity without clinical relevance [114].

Patients with suspected allergy to peanut, cashew, or hazelnuts should undergo measurement of specific IgE to Ara h 2, Ana o 3, and Cor a 14, respectively, in addition to SPT and IgE to extracts (Table 3 and Table 4) [104].

The Basophil Activation Test (BAT), which evaluates an allergen’s capacity to induce clinical symptoms of allergy by cross-linking specific IgE, which transduces a signal into the cell with the outcome of releasing of preformed mediators, is a promising adjunctive diagnostic tool, particularly in cases where traditional testing yields inconclusive results [125]. While traditional tests such as SPT and sIgE remain the foundation of diagnosis, BAT can provide supplementary information, particularly in unclear cases, and allow for a more individualized diagnostic approach in patients with atypical or inconsistent presentations of FA [126]. Some data indicate that BAT can reduce the need for OFC in patients sensitized to nuts [127]. BAT can be performed with either extracts or purified components, which may enhance test specificity but also carries a risk of misdiagnosis due to sensitization to non-clinically relevant components (Table 5) [124,126].

OFC remains the gold standard for confirming or excluding FA. However, it should be reserved for patients with unclear clinical history and inconclusive test results, after careful consideration of the risks and benefits. OFC is also used to qualify patients for allergen-specific immunotherapy, determine the starting allergen dose (eliciting dose), and monitor treatment efficacy. It must always be performed by an experienced medical team in accordance with safety protocols [104].

## 9. Treatment

The current standard of care for FA primarily involves strict allergen avoidance and prompt administration of emergency medications, such as intramuscular epinephrine, in the event of accidental exposure. Among available strategies, allergen-specific immunotherapy represents the only approach with the potential to modify the natural course of food allergy by inducing desensitization and, in some cases, long-term tolerance.

According to the European Academy of Allergy and Clinical Immunology (EAACI), oral immunotherapy (OIT) is recommended for selected patients with peanut, milk, and egg allergy [52,104,128]. Emerging evidence also supports the potential of epicutaneous (EPIT) and sublingual (SLIT) immunotherapy for peanut allergy, though these modalities remain under investigation [129,130]. In addition, OIT is currently being explored for the treatment of tree nut allergy, with preliminary studies suggesting possible benefit [131,132,133,134,135,136].

Despite its promise, allergen immunotherapy presents several challenges. Although effective, OIT carries a risk of adverse reactions, including systemic allergic responses and anaphylaxis. There is growing evidence that earlier initiation of immunotherapy may improve efficacy, yet further research is necessary to establish optimal timing, dosing protocols, long-term safety, and reliable biomarkers predictive of clinical response [137]. It has been proposed that combining different modalities, such as initiating treatment with SLIT or EPIT, followed by escalation to OIT, may enhance therapeutic outcomes while minimizing side effects [138].

Managing patients with multiple food allergies remains particularly complex. In 2024, the U.S. Food and Drug Administration (FDA) approved omalizumab, an anti-IgE monoclonal antibody, as an adjunctive treatment for food allergy in children over one year of age, in combination with allergen avoidance. Clinical studies suggest that omalizumab may also facilitate OIT by reducing adverse events, accelerating the achievement of maintenance dosing, and enabling desensitization to multiple allergens concurrently [139].

Finally, the use of immunomodulatory peptides as part of OIT protocols is an emerging area of investigation. These peptide-based approaches aim to improve the safety and efficacy of immunotherapy by selectively targeting allergen-specific immune responses and may represent a promising future direction in the treatment of food allergy [138,140].

## 10. Discussion

Peanut and tree nut allergies are among the most burdensome FA in childhood, both medically and psychosocially [141]. These IgE-mediated conditions can provoke severe and sometimes life-threatening anaphylactic reactions, requiring immediate administration of epinephrine [117]. The constant risk of accidental exposure significantly impacts quality of life, not only for affected children but also for their families, resulting in dietary limitations, social restrictions, and psychological stress [142].

Spontaneous resolution of peanut and tree nut allergies occurs in only a minority of patients, with approximately 20–34% of children outgrowing peanut allergy and even fewer achieving tolerance to tree nuts [80,99]. This limited potential for natural tolerance highlights the urgent need for effective primary prevention strategies and treatment.

Early dietary introduction of peanut has proven to be a highly effective preventive measure, particularly in Western populations, as demonstrated by the LEAP and EAT trial [51,53]. These landmark studies have shown that early oral exposure to peanuts in infancy significantly reduces the risk of allergy development. However, the generalizability of these results to other populations remains uncertain. In the Japanese JECS study, where the baseline risk is lower, early peanut introduction did not yield clear preventive benefits, suggesting that population-specific factors, including genetic and environmental exposures, may modulate the efficacy of prevention strategies [69]. Importantly, the global rise in food allergy cannot be explained by dietary practices alone. According to the epithelial barrier hypothesis, environmental factors such as pollution, detergents, and microbial exposures contribute to barrier impairment and subsequent allergic sensitization [143]. Thus, while early introduction of allergenic foods remains an important preventive measure, it is unlikely to be sufficient on its own, and broader strategies aimed at maintaining barrier integrity are needed.

Enhancing skin barrier function to prevent AD, a major risk factor for FA, has also been explored as a preventive approach. However, clinical trials using standard petroleum-based emollients (e.g., BEEP and PreventADALL) failed to demonstrate a protective effect against AD or FA [58]. Moreover, data suggest that emollient use in the general population may increase the risk of sensitization through transcutaneous allergen exposure [61]. In contrast, some studies using ceramide-rich emollients in high-risk infants have shown promise, underscoring the importance of tailored interventions and high-quality formulations [63]. These findings indicate that prevention of AD, and subsequent FA, may require more personalized strategies.

Emerging data support the value of identifying high-risk infants before clinical onset through genetic testing and biomarker profiling [144]. Genetic variants in *FLG*, *HLA-DQA1*, *IL13* as well as epigenetic markers and early immune and skin lipid signatures, have been associated with FA development [144,145,146]. The combination of these risk indicators with environmental exposures may enable stratification of infants most likely to benefit from early interventions, allowing for targeted surveillance and prevention.

Given the chronic nature of FA and the limited success and adverse effects associated with therapeutic interventions, such as OIT, there is increasing emphasis on prevention [52]. While OIT can induce desensitization and, in some cases, sustained tolerance, it is frequently associated with adverse reactions and increased epinephrine use compared to allergen avoidance [128]. EPIT offers a potentially safer alternative, with a more favorable safety profile, although its efficacy is still under investigation [135]. The combination of different immunotherapy modalities, as well as the concurrent use of omalizumab, has demonstrated a promising safety profile. Furthermore, novel peptide-based immunotherapy approaches may offer improved safety and efficacy, representing a potential advancement in the treatment of nut allergy.

For tree nut allergy, preventive data remain scarce. Preliminary findings suggest that early cashew introduction may reduce allergy risk, but these results are inconclusive and require validation in larger trials [57]. Once an allergy is established, OIT for nuts like cashew [133,134], hazelnut [132], and walnut [131] may be beneficial, yet robust comparative studies are still lacking. There is an urgent need for further research into preventive strategies that can be safely and effectively implemented across diverse populations to reduce the burden of tree nut allergy.

## 11. Conclusions

Peanut and tree nut allergies pose a significant burden due to their potential severity and impact on quality of life. As spontaneous resolution is uncommon, prevention remains a key priority. Early peanut introduction has proven effective, particularly in high-risk populations, though its applicability to low-risk groups requires further study. Identifying high-risk infants through genetic and biomarker profiling offers a promising avenue for targeted prevention. Education of parents and physicians is crucial to ensure successful implementation of preventive strategies. As treatment options remain limited and often carry risks, prioritizing safe and effective preventive strategies is essential to reduce the long-term burden of these allergies.

## Figures and Tables

**Table 1:** Summary of the different immune subtypes of nut-allergic patients.

Nut	Material	Distinct Immune or Barrier Features
Peanut [28]	Peripheral blood (B cells, T cells, DCs)	Peanut-allergic infants: ↑ CD19^high^HLA-DR^high^ B cells, ↑ TNF-α production; Peanut-sensitized but tolerant: ↑ plasmacytoid DCs, ↑ IL-2–producing naïve CD4^+^ T cells; Both allergic & tolerant: ↓ IFN-γ in effector memory T cells → impaired Th1 regulation
Peanut [29]	PBMCs	Upon peanut protein stimulation: ↑ MCP-1, MIP-1α, IL-6, IL-10; ↑ differentiation of CD11c^+^CD209^+^ DCs (IL-4/IL-13 dependent) with reciprocal Th2 interactions
Peanut/Tree nuts [30]	PBMCs (RNA-seq)	Transcriptomics: ↑ type I interferon signaling; ↓ humoral immunity; immune profile: ↑ neutrophils, ↓ regulatory T cells
Peanut (with AD) [31]	Non-lesional skin	↑ TEWL, ↓ filaggrin & sphingolipids, ↑ S. aureus colonization, ↑ keratins (K5, K14, K16); transcriptomics: enrichment of DC markers & Th2 pathways
Peanut (with AD) [32]	Non-lesional skin proteome—proteomic analysis	↑ inflammatory proteins (S100 family, alarmins, protease inhibitors), ↑ glycolytic enzymes, ↑ antioxidant defense proteins

↑ upregulation; ↓ downregulation.

**Table 2:** Summary of the prevalence of peanut and tree nut allergies in different world regions.

Nut (or Allergy Type)	Region/Country	Prevalence/Key Findings
Peanut	U.S., Canada, Australia	0.6–2% overall prevalence; ~1.6 million U.S. children affected
	U.S.	1.7%→5.2% (2001–2017) rise in annual incidence of peanut allergy in one-year-old individuals
	Europe (overall)	Lifetime prevalence 1.5%, point prevalence 2.1% (self-reported); challenge-confirmed ~0.1%
	UK	Increasing prevalence; in the UK, prevalence in all ages doubled 2001–2005 (0.024%→0.051%), incidence >2-fold increase in children 2000–2015 (0.116%→0.635%)
	Canada	Stable prevalence (2000–2007 in Montreal (1.34%→1.62%); increase in nationwide study 2010–2017 (2.7–3.5%)
	Australia	Increase from 0.73% (2001) to 1.15% (2004) in children aged 0–6 years
Tree Nuts (general)	U.S.	0.4–1.1%; ~0.9 million children affected
	Europe	0.03–8.5%; hazelnut most common (linked to birch pollen); pistachio least common
	Australia	Cashew most common; confirmed tree nut allergy up to 3.0% at age 6, 2.3% at age 10–14
	U.S. (FAAN study, >5000 individuals)	Walnut 34%, Cashew 20%, Almond 15%, Pecan 9%, Pistachio 7%; Hazelnut, Brazil nut, Macadamia < 5%
Peanut + Tree Nut Co-Allergy	U.S.	Sensitization to tree nuts in peanut-allergic: 65–86%, but clinical allergy only 20–34%
	UK	7.4% of children with peanut allergy also allergic to ≥1 tree nut
	Australia	30–40% of peanut-allergic children also allergic to tree nuts
	Central Europe (Poland)	35% of peanut-sensitized children are also sensitized to walnuts
Tree Nut Co-Allergy	U.S.	12–37% prevalence of multiple tree nut allergy
	Australia (HealthNuts)	47% of children with tree nut allergy allergic to >1 nut; 36% cashew-allergic also allergic to pistachio; Walnut–Pecan clustering less pronounced
	Israel (NutCracker study)	High clinical allergy among sensitized: Walnut 74.6%, Cashew 65.6%; strong clustering Walnut–Pecan, Cashew–Pistachio
	Europe (multicenter study)	60.7% allergic to >1 nut/seed; clustering Walnut–Pecan, Cashew–Pistachio, co-occurrence of Hazelnut and Macadamia allergy

**Table 3:** The most important proteins responsible for sensitization to peanuts. Bold font indicates the component of greatest clinical importance. The italic font represents the availability on commercial diagnostic platforms (based on Molecular Allergy User’s Guide 2.0 [124]).

Component	Protein Type	Clinical Function
*Ara h 1*	Seed-storage protein (7S globulin)	Major peanut allergen
***Ara h 2***	**Seed-storage protein (2S albumin)**	**Most predictive of clinical peanut allergy; responsible for most severe reactions**
*Ara h 3*	Seed-storage protein (11S globulin)	Major peanut allergen
Ara h 4	Isoform of Ara h 3	Potential major peanut allergen
Ara h 5	Profilin	Labile protein, not usually associated with severe reactions; associated with pollen sensitization (grass, birch, or sagebrush)
*Ara h 6*	Seed-storage protein (2S albumin)	Major peanut allergen, responsible for severe reactions
*Ara h 8*	Birch pollen homologue (Bet v1 homologue)	Labile protein, not usually associated with severe reactions; associated with pollen sensitization (birch)
*Ara h 9* Ara h 16 Ara h 17	Lipid-transfer proteins	Stable protein; associated with more severe symptoms in Mediterranean region especially with cofactors; associated with peach allergy
Ara h 10 Ara h 11 Ara h 14 *Ara h 15*	Oleosins	Stable proteins; associated with anaphylactic reactions, especially after consumption of roasted peanuts; hydrophobic allergens and are not present in the aqueous allergen extracts used for diagnosis; Isolated sensitization to oleosins is possible
Ara h 12 Ara h 13	Defensins	Stable proteins; associated with pollen sensitization (sagebrush); cross-reactions with sunflower (Hel a 4) and soybean (Gly m 2) allergens are possible.

**Table 4:** The most important proteins responsible for sensitization to tree nuts. Bold font indicates the component of greatest clinical importance. The italic font represents the availability on commercial diagnostic platforms (based on Molecular Allergy User’s Guide 2.0 [124].

Component	Protein Type	Clinical Role
Hazelnut		
*Cor a 1.04*	Birch pollen homologue (Bet v 1-homologue)	Labile protein, not usually associated with severe reactions; associated with pollen sensitization
Cor a 2	Profilin	Labile protein, not usually associated with severe reactions; associated with pollen sensitization (grass, birch or sagebrush)
*Cor a 8*	Lipid-transfer protein	Stable protein; associated with more-severe symptoms in Mediterranean region especially with cofactors; associated with peach allergy
***Cor a 9***	**Seed-storage protein (11S globulin)**	**Major hazelnut allergen**
Cor a 11 Cor a 16	Seed-storage protein (7S globulin)	Stable proteins; Associated with anaphylactic reactions
Cor a 12 Cor a 13 Cor a 15	Oleosins	Stable proteins; associated with anaphylactic reactions; hydrophobic allergens and are not present in the aqueous allergen extracts used for diagnosis; isolated sensitization to oleosins is possible;
Co a 14	Seed-storage protein (2S albumin)	Major hazelnut allergen; antibodies against Cor a 14 have the highest sensitivity, specificity, and predictive value in the diagnosis hazelnut allergy and monitoring of hazelnut tolerance
Walnut		
***Jug r 1***	**Seed-storage protein (2S albumin)**	**Major walnut allergen; associated with anaphylactic reactions; shows extremely high (87%) homology with the phylogenetically related pecan allergen Car i 1 and black walnut (Jug n 1) and slightly lower (66%) with Cor a 14 from hazelnut**
*Jug r 2*	Seed-storage protein (7S globulin)	Shows a high degree of cross-reactivity with the major peanut allergen Ara h 2 despite the low homology of both allergens.
*Jug r 3* Jug r 8	Lipid-transfer protein	Stable protein; associated with more severe symptoms in Mediterranean region especially with cofactors; associated with peach allergy
*Jug r 4*	Seed-storage protein (11S globulin)	Minor walnut allergen; sIgE against Jug r 4 has a very high (90%) positive predictive value for allergic symptoms after walnut consumption, Jug r 4 is highly homologous to pecan 11S globulin (95%)
*Jug r 6*	Seed-storage protein (7S globulin)	Shows a high degree of homology with 7S globulins from hazelnut Cor a 11 (76%), pistachio (62%) and sesame (58%)
Jug r 7	Profilin	Labile protein, not usually associated with severe reactions; associated with pollen sensitization (grass, birch or sagebrush)
Cashew		
Ana o 1	Seed-storage protein (7S globulin)	Highly homologous to Pis v 3 in pistachio nut, Cor a 11 in hazelnut and Jug r 2 in walnut
*Ana o 2*	Seed-storage protein (11S globulin)	Highly homologous to Pis v 5 in pistachio; high similarity of the conformational epitope of Ara h 3 from peanuts has also been demonstrated, despite low primary sequence homology; cross-reactions with Cor a 9 (hazelnut) and Jug r 4 (walnut) are also possible.
***Ana o 3***	**Seed-storage protein (2S albumin)**	**Major cashew allergen; associated with severe allergic reaction; monosensitization to Ana o 3 is associated with the most severe anaphylactic reactions; responsible for cross reactions with pistachio (Pis v 1), sesame (Ses i 1), walnut (Jug r 1), allergens found in citrus fruit seeds (oranges, mandarins), Sichuan pepper, pomegranates and mangoes.**
Pistachio nut		
*Pis v 1*	Seed-storage protein (2S albumin)	Major pistachio nut allergen; homologue to Ana o 3
*Pis v 2* Pis v 5	Seed-storage proteins (11S globulins)	Major pistachio nut allergens; homologues to Ana o 2
*Pis v 3*	Seed-storage protein (7S globulin)	Homologue to Ana o 1
Almond		
Pru du 1	Birch pollen homologue (Bet v 1-homologue)	Labile protein, not usually associated with severe reactions; associated with pollen sensitization
Pru du 3	Lipid transfer protein	Stable protein; associated with more-severe symptoms in Mediterranean region especially with cofactors; associated with peach allergy
Pru du 4	Profilin	Labile protein, not usually associated with severe reactions; associated with pollen sensitization (grass, birch or sagebrush)
Pru du 6 (Amandin)	Seed-storage protein (11S globulin)	Major almond allergen; associated with severe reactions
Pecan		
Car i 1	Seed-storage protein (2S albumin)	Shows extremely high (87%) homology with the phylogenetically related walnut allergen Jug r 1
Car i 2	Seed-storage protein (7S globulin)	Homologue to Jug r 2
Car i 4	Seed-storage protein (11S globulin)	Shows extremely high (95%) homology with the phylogenetically related walnut allergen Jug r 4

**Table 5:** Sensitivity and specificity of different diagnostic methods for peanut and tree nut sensitization evaluation. Based on EAACI Guidelines on diagnosis of IgE-mediated food allergy [104,126].

Diagnostic Test	Peanut	Cashew	Hazelnut
Skin prick test			
Cut-offs (mm)	4 (3–8)	5 (4–6)	5 (3–7)
Sensitivity	0.84 (0.69–0.92)	0.93 (0.89–0.96)	0.82 (0.68–0.91)
Specificity	0.86 (0.79–0.91)	0.92 (0.82; 0.96)	0.78 (0.44–0.94)
Specific IgE of allergen extracts			
Cut-offs (kU/L)	4.3 (0.35–10)	1.1 (0.6–3.1)	2.34 (0.6–6.3)
Sensitivity	0.81 (0.71–0.88)	0.94 (0.89–0.97)	0.79 (0.71–0.85)
Specificity	0.83 (0.71–0.90)	0.64 (0.54–0.74)	0.62 (0.38–0.81)
Specific IgE of allergen-components			
Cut-offs (kU/L)	Ara h 2 0.44 (0.3–1.3)	Ana o 3 0.4 (0.2–0.6)	Cor a 14 0.64 (0.35–3.5)
Sensitivity	0.82 (0.77–0.86)	0.96 (0.91–0.98)	0.73 (0.53–0.87)
Specificity	0.92 (0.87–0.95)	0.94 (0.88–0.97)	0.95 (0.90–0.98)
Basophil activation test			
Cut-offs (%CD63^+^ Basophils)	5.0 (4.7–7.1)	-	-
Sensitivity	0.84 (0.76–0.90)	-	-
Specificity	0.90 (0.83–0.94)	-	-

Values in brackets indicate 95% CI; specific IgE of extracts and specific IgE of allergen-components were measured using ImmunoCAP; there are no established cut offs of BAT results in cashew and hazelnut allergy diagnosis.

## Data Availability

There was no new data created for the purpose of this article.

## Abbreviations

The following abbreviations are used in this manuscript:

AD	atopic dermatitis
BAT	basophil activation test
CRD	component-resolved diagnostics
EAT	Enquiring About Tolerance study
EPIT	epicutaneous immunotherapy
FA	food allergy
(s)IgE	(specific) immunoglobulin E
IL	interleukin
LEAP	Learning Early About Peanut Allergy study
LTPs	lipid transfer proteins
OIT	oral immunotherapy
PAS	Peanut Allergy Sensitization
PFAS	pollen food allergy syndrome
SLIT	sublingual immunotherapy
SPT	skin prick test
TEWL	transepidermal water loss
UK	United Kingdom
U.S.	United States
